# Comparative Evaluation of Accuracy of Adjacent Parallel Implant Placements Between Dynamic Navigation and Static Guide: A Prospective Study

**DOI:** 10.7759/cureus.57331

**Published:** 2024-03-31

**Authors:** Dnyaneshwar Parekar, Sahana Selvaganesh, Thiyaneswaran Nesappan

**Affiliations:** 1 Implantology, Saveetha Dental College, Saveetha Institute of Medical and Technical Sciences, Chennai, IND; 2 Prosthodontics and Implantology, Saveetha Dental College, Saveetha Institute of Medical and Technical Sciences, Chennai, IND

**Keywords:** accuracy, surgical guides, cone beam computed tomography, guided implant surgery, dynamic navigation

## Abstract

Aim

The study aims to compare the accuracy of dynamic navigation (DN) and static guides (SGs) for simultaneous adjacent parallel placement of implants, the time taken for the surgery, and the ease of handling the instruments.

Materials and methods

This prospective trial was carried out at the Department of Implantology of Saveetha Dental College from October 2022 to February 2023. A total of 20 patients who needed simultaneous adjacent dental implants were allocated randomly into two groups: Group 1 SG surgery and Group 2 DN surgery. Forty implants were placed, 20 under DN and 20 under SG. Bucco-lingual displacement, apico-coronal displacement, mesiodistal displacement, and mesiodistal angulation were compared between the two groups. The patients’ data in both groups were evaluated by operating surgeons along with the surgical time taken and the ease of handling of instruments.

Results

The 20 patients who underwent implant placement in the DN and SG groups all had adjacent missing teeth in posterior sites, including lower posteriors (70%) and upper posteriors (30%). There was improved precision in relation to the mesiodistal displacement and angulation of the anterior implant of the adjacent parallel implants. The mesiodistal displacement in Group 1 (SG) was 5.61 + 3.1 mm, which was higher than Group 2 (DN), which was 0.55 + 0.56 mm. The mesiodistal angulation was 3.1 + 2.9 degrees in Group 2 and 0.42 + 0.5 degrees in Group 1. The second implant had a significant difference (p < 0.005) in mesiodistal displacement, mesiodistal angulation, and bucco-lingual displacement. The difference between the intergroup surgical time (mean + SD) in Group 1 was 30 + 4.5 mins and in Group 2 was 60.7 + 10.1 mins, with p < 0.05 statistically significant. The comfort of the operator was better in the SG group.

Conclusion

Any digitally aided implant placement technique can improve placement accuracy but each has its downfalls. Achieving the highest levels of precision and long-lasting prosthetic results hinges on both the suitability of the chosen case and the expertise of the surgeon performing the implant placement.

## Introduction

Over the past 10 years, dental implants have gained much popularity in terms of their success and survival with more and more edentulous patients opting for this method. This can be attributed to the natural appearance that the implants provide in the long run. The success and survival of the implants rely on the correct placement and type of prosthesis that is provided on top of the implant. This boils down to the fact that the implant placement positions need to be preplanned (WG1) prior to the surgical appointment. Patients requiring dental implant placements must have a streamlined appointment. Several other factors can play a major role in the success of the implant, such as anatomical limitations in the maxilla, bone density, wound healing, and crestal bone loss in the long run [1-4].

Dynamic navigation (DN) is a digital computer-aided implant surgery technique that uses advanced computer technology to help navigate the implants to the correct preplanned path. It provides real-time feedback to the dental surgeon during the implant placement procedure, allowing for greater precision and accuracy [5,6].

The Navident (Claronav, Canada) system uses a series of instruments for sequential calibration and merging of the preloaded patient’s data to the real-time patient. For this purpose, Navident uses a calibrator and a tracer, which are hand-held devices. This device is used to trace the patient’s mouth, creating a 3D map of the teeth and surrounding structures and matching it to the cone beam computed tomography (CBCT) data already loaded during the treatment planning phase. The dental surgeon can use this virtual model to plan the placement of the dental implant, taking into account important considerations such as the density of bone, implant size, and the location of important structures such as nerves and blood vessels. During the actual implant placement procedure, the Navident system uses real-time feedback to guide the dental surgeon. The handheld device is employed to track the position of the implant and monitor its placement, ensuring that it is placed in the correct position and at the correct angle [7,8].

The Navident system can be used for a variety of cases pertaining to dental implantology, including single-tooth implants, multiple-tooth implants, full arch restorations, and complex surgical procedures such as sinus lifts. It is particularly useful for complex cases in which the location of important structures may be difficult to determine [9].

Guided implant placement is a computerized digitally aided placement technique that involves the use of a surgical guide or template. The surgical template is created based on a 3D model of the patient's mouth, which is usually generated using digital imaging technologies such as CBCT or intraoral scanners [6,9,10]. The surgical guide is a custom-made device that fits over the patient's teeth or gums and provides a precise guide for the implant placement. The guide has pre-planned holes that correspond to the desired implant locations, angles, and depths [11].

During the implant placement procedure, the surgical guide is positioned in the patient's mouth, and the dentist uses it to precisely place the dental implant(s). The guide ensures that the implants are placed in the correct position and orientation, according to the pre-planned design. This can help reduce the risk of errors and complications that can occur with freehand implant placement [10-12].

Although some studies have begun to explore the accuracy of implant placement with DN compared to static surgical guides for implant placement, the existing research landscape remains limited, with most investigations confined to in vitro studies [13-15]. A study by Wu et al. suggested that there was no statistically significant difference between the two groups. However, in his study, the apical deviation of the DN was slightly higher than the static guide (SG) group in the anterior teeth (p = 0.028), and the angular deviation of DN was smaller than the SG group in the molar region [16]. The primary objective was to evaluate the clinical accuracy of DN by measuring coronal, apical, and angular deviations during implant placement. Secondary objectives were to assess the time taken for the procedure between the groups, and we also sought to analyze the potential influence of user-related factors such as ease of use and instrumentation. This focus on user experience distinguishes our study from previous efforts, which often neglected the potentially significant impact of operator interaction with the respective guidance systems.

Null hypothesis: There is no significant difference between the accuracy of the implant placement between the static guidance and dynamic navigation systems.

Alternate hypothesis: There is a significant difference between the accuracy of the implant placement between the static guidance and dynamic navigation systems.

## Materials and methods

This study was a single-center, parallel-group, prospective study. The study was cleared by the institutional review board with the ethical clearance number (1904/22/017). The study was conducted from October 2022 to February 2023 in the Department of Oral Implantology at Saveetha Dental College, Chennai, India. A total of 20 patients were selected based on specific criteria. Participants included were at least 20 years old, requiring the placement of two adjacent implants, maintaining good oral hygiene, adequate bone width and density, and not experiencing significant anxiety associated with dental procedures or surgical instruments (especially while DN is laced in the operating theater (OT)) [17]. Patients requiring additional bone grafting procedures or full mouth rehabilitation were excluded. These eligible individuals were then randomly divided into two groups using block randomization: Group 1 received guided surgery with pre-planned guides, and Group 2 underwent Navident-assisted surgery employing real-time navigation for precise implant placement. Both groups followed the same standard dental implant placement protocol.

Group 1: SG - working protocol

**Figure 1:**
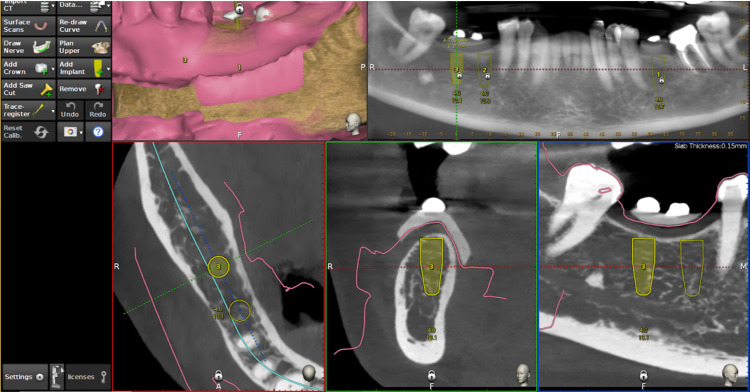
Dynamic navigation - STL and DICOM files merging and implant positional planning DICOM: digital imaging and communications in medicine; STL: stereolithography

Patients in this group were subjected to routine radiographic analysis and intraoral scanning, and the DICOM (digital imaging and communications in medicine) files were combined with the STL (stereolithography) files from intraoral scans using the DN Navident software for implant planning and positioning [18] (Figure 1).

**Figure 2:**
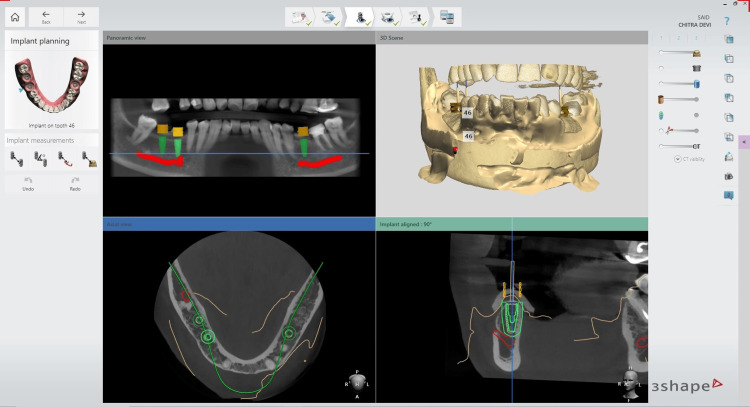
Exported .stl from Navident software and implant planning in 3Shape software

The planning of the precise implant positions was carried out by the operator. The planned file was then exported as a .stl. This exported file was further designed using 3Shape (Copenhagen, Denmark) software (Figure 2). The operator, at this stage, assessed the accuracy and congruence between the two planned positions.

**Figure 3:**
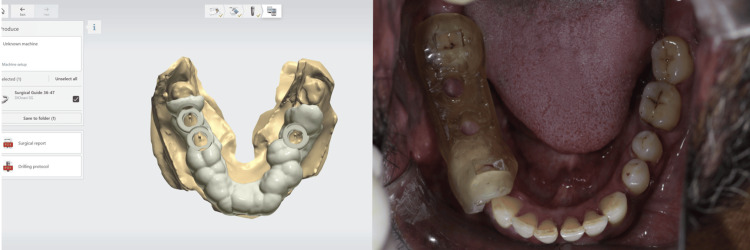
Guide designing, printing, and intra-oral seating verification

Once the final implant positions were confirmed, the static guide design process commenced. This design was then transformed into a physical guide using a DIO PROBO 3D printer (Busan, South Korea). After printing, the guide was cured and sterilized for subsequent use (Figure 3). On the day of surgery, sequential drilling was carried out and the implants were placed [19].

Group 2: DN - working protocol

Patients in the DN groups were also subjected to the same radiographic analysis and scanning protocol. The positional implant planning was carried out with the DN software Navident (Figure 1). During the surgical appointment, calibration and setting up of the machine was done in the OT. Prior to surgery, within the sterile OT, a crucial step ensures precise implant placement - calibrating and orienting the Navident system cameras to the patient and their surroundings. This involves several key procedures:

1. Drill tag and handpiece calibration

● Drill tags with optical sensors are attached to the handpiece

● Using the camera and a quick response (QR) code on the drill tags, the handpiece calibration is performed on the computer

● This ensures the DN cameras can continuously track the handpiece's position throughout the surgery

2. Patient orientation and calibration

● Depending on the jaw being operated on, the patient's orientation is established:

- Maxillary implants: head trackers are used 

- Mandibular implants: jaw tracers are used

● The tracers are positioned on stable points identified on either the DICOM data (X-rays) or the STL data (scan)

● These points are then clinically correlated with the tracer tip's position in the patient's mouth

● To further enhance accuracy, the entire tooth surface is traced, ensuring seamless merging of clinical and diagnostic data. 

Accuracy analysis

**Figure 4:**
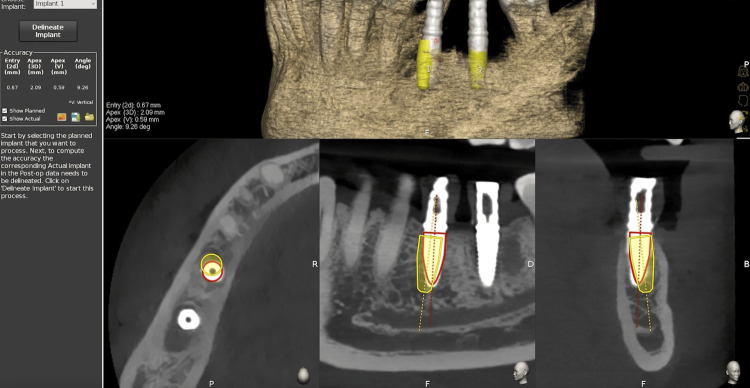
The EvaluNav analysis and CBCT merging CBCT: cone beam computed tomography

**Figure 5:**
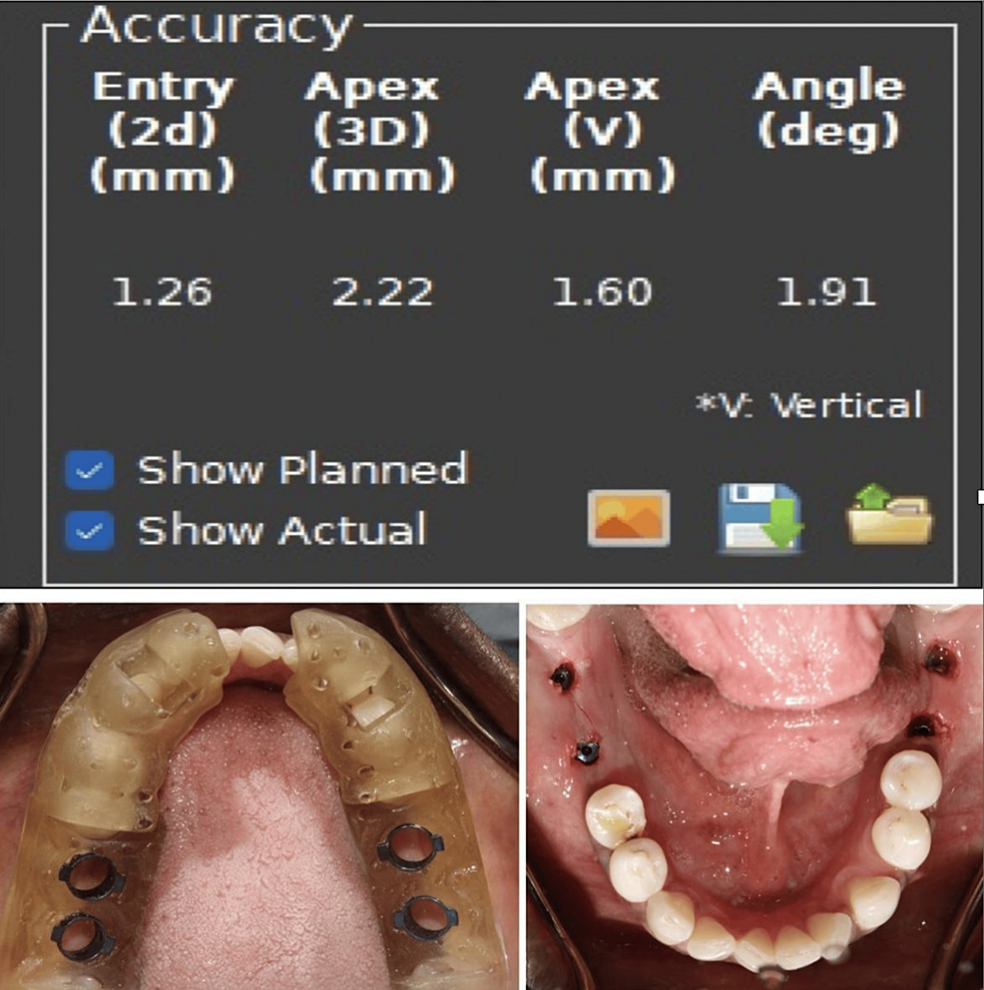
Implant placement using guides and accuracy analysis with EvaluNav software

After the implant placement, the patients were subjected to CBCT analysis, which was used to assess the accuracy of bucco-lingual displacement, apico-coronal displacement, mesiodistal displacement, and mesiodistal angulation. The planning and postplacement CBCT were merged using the EvaluNav application of the Navident software (Figures 4-5). The CBCT data were then merged, the planned implant position was correlated with the placed implant position, and the deviation in values was noted down.

Surgical time

The time taken for the surgery was noted with a stopwatch kept in the surgical OT. The time was noted beginning from the setting up, calibration, and placement for the DN group and from the seating verification and drilling protocol for the SG group till the implant placement. No suturing was involved, and the patients were recalled after a week for a review and were then followed up till the third month after the implant placement. Later, final prostheses were given [20].

Ease of use and instrumentation

Beyond the technical accuracy of implant placement techniques, ensuring the surgeon's comfort and ease of operation is crucial for successful outcomes. This study utilized a questionnaire with Likert scale marking to evaluate and compare the operator's experience with both DN and SG approaches for each case (Table 1) [21].

**Table 1:** Questionnaire used to assess the workability and instrumentation

Questions	Likert Scale
The instrument is lightweight and readily passed to the operator	Strongly Disagree/Disagree/Neutral/Agree/Strongly Agree
Calibration and accuracy of the devices are easily verified	Strongly Disagree/Disagree/Neutral/Agree/Strongly Agree
The device/instruments restrict the movement of the operator inside the OT	Strongly Disagree/Disagree/Neutral/Agree/Strongly Agree
Hand-eye coordination has an impact on the accuracy of that particular case	Strongly Disagree/Disagree/Neutral/Agree/Strongly Agree
The instrumentation is easier in cases with restricted mouth-opening	Strongly Disagree/Disagree/Neutral/Agree/Strongly Agree

## Results

Twenty participants with a mean age of 43 years were considered for the study. Forty implants placed were evaluated. The subjects included had edentulous sites, lower posteriors (70%), and upper posteriors (30%) with respect to adjacent parallel implants. A Mann-Whitney U test was carried out to assess the accuracy between the two groups.

First Implant

Among the 20 patients who underwent implant placement under DN and SG, there was a significant difference (p < 0.005) in mesiodistal displacement and mesiodistal angulation with respect to the first implant among the parallel implants (Table 2). The mesiodistal displacement in Group 1 (SG) of 5.61 + 3.1 mm was higher than Group 2 (DN), which had 0.55 + 0.56 mm. The mesiodistal angulation was 3.1 + 2.9 (degree) in Group 2 and 0.42 + 0.5 (degree) in Group 1.

**Table 2:** Mean, standard deviation, and significance of various accuracy parameters for the first implant among the adjacent parallel implants placed Accuracy analysis for the first implant placed among the adjacent parallel implants. *Significant difference p < 0.05

Accuracy Parameters	Dynamic Navigation (Mean+ SD)	Static Guidance (Mean+ SD)	Significance
Mesiodistal Displacement	0.55 + 0.56 mm	5.61 + 3.1 mm	0.01*
Mesiodistal Angulation	0.42 + 0.5 (degree)	3.1 + 2.9 (degree)	0.00*
Bucco-lingual Displacement	0.29 + 0.33 mm	0.9 + 0.4 mm	0.20
Apico-coronal Displacement	0.88 + 0.51 mm	1.2 + 0.8 mm	0.43

Second Implant

The second implant had a significant difference (p < 0.005) in mesiodistal displacement, mesiodistal angulation, and bucco-lingual displacement (Table 3). The mesiodistal displacement with the SG group was the highest at 5.53 + 2.93 mm.

**Table 3:** Mean, standard deviation, and significance of various accuracy parameters for the second implant among the adjacent parallel implants placed Accuracy analysis of the second implant placed among the adjacent parallel implants. *Significant difference p < 0.05

Accuracy Parameters	Dynamic Navigation (Mean+ SD)	Static Guidance (Mean+ SD)	Significance
Mesiodistal Displacement	2.52 + 2.02 mm	5.53 + 2.93 mm	0.02*
Mesiodistal Angulation	1.1 + 0.5 (degree)	2 + 1.2 (degree)	0.01*
Bucco-lingual Displacement	1.31 + 0.34 mm	0.9 + 0.4 mm	0.06*
Apico-coronal Displacement	0.9 + 0.6 mm	1.14 + 0.8 mm	0.59

A significant difference in the surgical time taken between the two groups was noted (mean time Group 1 = 30 + 4.5 mins, Group 2 = 60.7 + 10.1 mins) with p < 0.05.

The workability and instrumentation were assessed with the questionnaire described in Table 1. The results of the same are given below.

Question 1: The instrument is lightweight and readily passed to the operator

● Group 1: 100% agree

● Group 2: 100% disagree

Interpretation: The operator for all the cases in Group 1 found the instrument easy to handle and pass, whereas in Group 2 the operator disagreed. This suggests a significant difference in the perceived ease of instrument exchange between the two groups. The DN system involves the use of many hand-held instruments such as the trace register, calibrator, jaw trackers, and drill tags attached to the operating handpiece. The workability and the weights of all these vary, altering the comfort of the operating surgeon.

 Question 2: Calibration and accuracy of the devices are easily verified

● Group 1: Not applicable 

● Group 2: 40% strongly disagree, 60% agree

Interpretation: For Group 1 there is no calibration involved. However, in Group 2, for the majority of cases the operator agreed that calibration and accuracy were easy to verify, but for notable cases the operator still disagreed. This indicates potential room for improvement in the verification process. The calibration process with the DN unit involves time; this forms a learning curve where the operating surgeon learns to improve their calibration and accuracy assessment with each case and there has been a steady increase in the same. The calibration accuracy is not in all cases attained in the first go unless the surgeon is well trained in using the DN system.

Question 3: The device/instruments restrict the movement of the operator inside the OT

● Group 1: 100% strongly disagree

● Group 2: 100% strongly agree

Interpretation: This shows a stark contrast between the groups. Operators for all cases in Group 1 felt unrestricted in their movements. While the operator for all cases in Group 2 found it limiting. This could be a crucial factor in choosing between the two options based on surgeon preference and comfort.

Question 4: Hand-eye coordination has an impact on the accuracy of that particular case

● Group 1: Not applicable 

● Group 2: 100% agree

Interpretation: Group 2 participants unanimously agreed that hand-eye coordination affects accuracy. This highlights the importance of manual dexterity and skill in achieving precise implant placement regardless of the chosen technique. This can also be attributed to the learning curve associated with DN.

Question 5: The instrumentation is easier in cases with restricted mouth-opening

● Group 1: 30% disagree, 70% agree

● Group 2: 50% disagree, 50% neutral

Interpretation: Group 1 shows the operator majorly agreeing that the instrumentation is easier with limited mouth-opening, though for some cases he still disagreed. Interestingly, Group 2 was evenly divided: half disagreed while the other half remained neutral. This suggests different opinions on the instrument's ease of use in each situation.

## Discussion

The findings of this study reject the null hypothesis, which suggests that there is no significant difference between the accuracy of the implant placement between the two systems of guidance. The results suggest that the DN has an upper hand in establishing accurate mesiodistal displacement and angulation compared to the SG system. Some previous studies have suggested that there can be drawbacks to the SG system. The literature review by Unsal et al. revealed a critical point: practitioners, especially the inexperienced ones, must be informed about the potential angular and linear deviations of up to 5 degrees and 2.3 millimeters when using computer-aided designed and computer-aided manufactured (CAD/CAM) surgical guides. Adequate training and familiarity with the basic steps are crucial to mitigate risks and avoid complications [22]. Another systematic review by Wu et al. analyzed data from various clinical trials, suggesting that the drawbacks of the SG system could be attributed to various aspects such as planning, errors in 3D printing, guide positioning, guide fixation, and type of the guide chosen for the particular surgical procedure. The systematic review revealed a mean deviation at the entry point of 1.25 mm (95% confidence interval (CI): 1.22-1.29), 1.57 mm (95% CI: 1.53-1.62) at the apex, and 4.1° in angle (95% CI: 3.97-4.23) [16]. This disparity in the results of this study and the previous studies based on static and dynamic navigation is the planning gap between the DN and SG systems. The compatibility between the two software can be a potential limitation. The errors that might arise because of the planning and accuracy checking can be reduced by designing software that is compatible with both the techniques of static and dynamic navigation.

The software enables computer-assisted implant planning and surgery based on computer-aided design by combining data from the CBCT scanner's DICOM files in a virtual environment with data from the intraoral scanner's STL files. Currently, there is little research comparing the time and cost of dynamic navigation versus static navigation. However, current evidence indicates that DN is significantly more expensive than other approaches. It's also worth remembering that as technology advances, so do the costs, and that such devices aren't yet widely used.

Surgical guides are custom-made based on these scans, ensuring that the implant is placed in the intended location and angle. This accuracy often leads to better implant success rates, reduced postoperative discomfort, and improved overall patient satisfaction [23]. This also provides more comfort for the operators in instrumentation and handling in comparison with the DN system. However, it's important to note that the accuracy can also be influenced by factors like the quality of the CT scan, the design of the surgical guide, and the dentist's skill and experience.

Narrowing down on DN or static guiding for implant placement will require further study. Trace registration technology offers a fully digital process. It cuts down the time the patient invests for a second CBCT with fiducial markers and also reduces the technique-sensitive step of constructing a custom stent before the surgical appointment. DN's effectiveness and applicability have risen thanks to this technology. The most important benefit of DN is that, unlike a static technique, it enables continual accuracy checks throughout the operation. The real-time guidance of DN can potentially accelerate the implant placement stage due to its precise visual feedback and adjustments made throughout the procedure. These findings of the study indicate that there is a significant difference in the mesiodistal displacement and angulation between the two guidance methods. This can be attributed to the material of choice of the surgical resin and the shrinkage of the guides post-printing. There were problems associated with the seating of guides in a few cases and there was a loss of the surgical guides (breakage) after sequential drilling; all these can be confounding factors that could have hampered the results. 

The learning curve associated with the DN technique is steep. The operator who performed this study had an improved experience with the DN system. The non-significant results associated with the apico-coronal displacement and bucco-lingual displacement can be attributed to the instrumentation and the restriction that DN provides. Particularly, the surgeon is expected to work looking at the screen with improved hand-eye coordination, as the weight of the handpiece can deviate the path of drilling if the haptic feedback of the surgeon is not up to the mark [24]. The results of this study correlate with the findings of Chen et al., in which there were increased mesiodistal and angular deviations with the SG than the DN systems, 1.35 ± 0.55 mm was the mean horizontal deviation at the apical endpoint when using DN system, 1.50 ± 0.79 mm with static navigation system, and higher angular deviation values were reported for static navigation system (6.02 ± 3.71°) than for the DN system (4.45 ± 1.97°) [25]. The success and the accuracy of the implant placement greatly depend on the designing, planning, and template fabrication process; if there are any inaccuracies in this step, it can lead to more deviations. With DN, there is a constant need for the operators to be looking at the screen and a lot of information about the surgical site gets lost, whereas with SG, the operators can look at the surgical site constantly [15]. Augmented reality could be an alternative to provide a simultaneous view of the navigation screen and the surgical field [26,27].

By analyzing the time taken for each specific stage, we gain a deeper understanding of the strengths and limitations of both DN and SG. This information can then be used to tailor treatment plans for individual patients, ensuring optimal efficiency and successful outcomes. The surgical time required for DN is longer because it involves the calibration process, but this technique can be more efficient in patients who are taking anticoagulant medications for systematic disorders such as cardiovascular ailments, where the surgical site can be made as minimally invasive as possible and suturing can be avoided. DN guidance methods could improve the accuracy of implant placement more, but given the learning curve, weight of the handpiece, surgical time, and calibration process, SGs can provide a quicker route with improved accuracy. Considering the drawbacks of both groups, further research is required to assess the accuracy of implant placement with a particular system, along with their potential complications.

## Conclusions

Acknowledging the boundaries and the limitations of this study, the DN system seems to perform better in terms of mesiodistal displacement and angulation when compared to the SG system in adjacent implant placement sites, whereas they showed similar accuracy in bucco-lingual and apico-coronal deviations, which makes both systems reliable. Free-hand drilling can provide tactile sense and direct visualization of the bone and soft tissues, and adding DN can facilitate visualization of the drill going through the bone.

The procedures can be done flapless, and there is scope for correction of the position of the implants mid-way during the surgery with DN. This can provide the patient with improved comfort, lessen the post-operative healing time, and render an overall successful experience for both the operator and the patient. Further clinical trials with an improved sample size are required to analyze the real behavior and usability of both computer-assisted navigation systems.
